# Haldane’s formula in Cannings models: the case of moderately strong selection

**DOI:** 10.1007/s00285-021-01698-9

**Published:** 2021-12-06

**Authors:** Florin Boenkost, Adrián González Casanova, Cornelia Pokalyuk, Anton Wakolbinger

**Affiliations:** 1 Goethe-Universität Frankfurt, FB 12, 60054 Frankfurt, Germany; 2 Instituto de Matemáticas, Universidad Nacional Autónoma de México, Área de la Investigación Científica, Circuito Exterior, Ciudad Universitaria, 04510 Coyoacán, CDMX, Mexico

**Keywords:** Branching process approximation, Cannings model, Directional selection, Probability of fixation, Primary 60J10, Secondary 60J80, 92D15, 92D25

## Abstract

For a class of Cannings models we prove Haldane’s formula, $\pi (s_N) \sim \frac{2s_N}{\rho ^2}$, for the fixation probability of a single beneficial mutant in the limit of large population size *N* and in the regime of moderately strong selection, i.e. for $s_N \sim N^{-b}$ and $0< b<1/2$. Here, $s_N$ is the selective advantage of an individual carrying the beneficial type, and $\rho ^2$ is the (asymptotic) offspring variance. Our assumptions on the reproduction mechanism allow for a coupling of the beneficial allele’s frequency process with slightly supercritical Galton–Watson processes in the early phase of fixation.

## Introduction

Analysing the probability of fixation of a beneficial allele that arises from a single mutant is one of the classical problems in population genetics, see Patwa and Wahl (2008) for a historical overview. A rule of thumb known as Haldane’s formula states that the probability of fixation of a single mutant of beneficial type with small selective advantage $s > 0$ and offspring variance $\rho ^2$ in a large population of individuals, whose total number *N* is constant over the generations, is approximately equal to $2s/\rho ^2$. Originally, this was formulated for the (prototypical) model of Wright and Fisher, in which the next generation arises by a multinomial sampling from the previous one (which leads to $\rho ^2 = 1-\frac{1}{N}$ in the neutral case), with the “reproductive weight” of an individual of beneficial type being increased by the (small) factor $1+s$. A natural generalization of the Wright-Fisher model are the Cannings models; here one assumes *exchangeable* offspring numbers in the neutral case (Cannings 1974; Ewens 2004), and separately within the sets of all individuals of the beneficial and the non-beneficial type in the selective case (Lessard and Ladret 2007).

The reasoning in the pioneering papers by Fisher (1922), Haldane (1927) and Wright (1931) was based on the insight that, as long as the beneficial type is rare, the number of individuals carrying the beneficial type is a slightly supercritical branching process for which the survival probability is

$$\begin{aligned} \pi (s) {\sim } \frac{2s}{\rho ^2} \qquad \text { as } s \rightarrow 0, \end{aligned}$$

where $1+s$ is the offspring expectation and $\rho ^2$ is the offspring variance (see Athreya 1992, Theorem 3). The heuristics then is that the branching process approximation should be valid until the beneficial allele has either died out or has reached a fraction of the population that is substantial enough so that the law of large numbers dictates that this fraction should rise to 1.

Notably, Lessard and Ladret (2007) obtained (for fixed population size *N*) the result

1 $$\begin{aligned} \pi (s) = \frac{1}{N} + \frac{2s}{\rho ^2} + o(s) \qquad \text { as } s \rightarrow 0, \end{aligned}$$

as a special case of their explicit analytic representation of $\pi (s)$ within a quite general class of Cannings models and selection mechanisms.

An interesting parameter regime as $N\rightarrow \infty$ is that of *moderate selection*,

2 $$\begin{aligned} s_N \sim c N^{-b} \quad \text{ with } \quad 0<b<1, { \quad c>0}, \end{aligned}$$

which is between the classical regimes of *weak* and *strong* selection. Is the *Haldane asymptotics*

3 $$\begin{aligned} \pi (s_N) \sim \frac{2s_N}{\rho ^2}\qquad \text { as } N \rightarrow { \infty }, \end{aligned}$$

valid in the regime (2)?

If one could bound in this regime the *o*(*s*)-term in (1) by $o( N^{-b})$, then (1) would turn into (3). Such an estimate seems, however, hard to achieve in the analytic framework of Lessard and Ladret (2007).

The main result of the present paper is a proof of the Haldane asymptotics using an approximation by Galton–Watson processes in the regime of *moderately strong selection*, which corresponds to (2) for $0< b < \frac{1}{2}$. Hereby, we assume that the Cannings dynamics admits a *paintbox representation*, whose random weights are exchangeable and *of Dirichlet-type*, and fulfil a certain moment condition, see Sect. 3. Here, the effect of selection is achieved by a decrease of the reproductive weights of the non-beneficial individuals by the factor $1-s_N$.

An approximation by Galton–Watson processes was used in González Casanova et al. (2017) to prove the asymptotics (3) in the regime of moderately strong selection for a specific Cannings model that arises in the context of experimental evolution, with the next generation being formed by sampling without replacement from a pool of offspring generated by the parents.

In the case $b \ge \frac{1}{2}$ the method developed in the present paper would fail, because then the Galton–Watson approximation would be controllable only up to a time at which the fluctuations of the beneficial allele (that are caused by the resampling) still dominate the trend that is induced by the selective advantage. However, in Boenkost et al. (2021) we proved the Haldane asymptotics (3) for the case of *moderately weak selection*, i.e. under Assumption (2) with $\frac{1}{2}< b <1$. There a backward point of view turned out to be helpful, which uses a representation of the fixation probability in terms of sampling duality via the *Cannings ancestral selection graph* developed in Boenkost et al. (2021) (see also González Casanova and Spanó 2018).

The results of the present paper together with those of Boenkost et al. (2021) do not cover the boundary case $b=\frac{1}{2}$ between moderately strong and moderately weak selection. We conjecture that the Haldane asymptotics (3) is valid also in this case.

## A class of Cannings models with selection

This section is a short recap of Boenkost et al. (2021) Section 2; we include it here for self-containedness.

### Paintbox representation in the neutral case

Neutral Cannings models are characterized by the exchangeable distribution of the vector $\nu =(\nu _1,\dots , \nu _N)$ of offspring sizes; here the $\nu _i$ are non-negative integer-valued random variables which sum to *N*. An important subclass are the *mixed multinomial* Cannings models. Their offspring size vector $\nu$ arises in a two-step manner: first, a vector of random weights ${\mathscr {W}}=(W_1,\dots ,W_N)$ is sampled, which is exchangeable and satisfies $W_1+\cdots +W_N=1$ and $W_i\ge 0$, $1 \le i \le N$.

In the second step, a die with *N* possible outcomes $1,\ldots , N$ and outcome probabilities ${\mathscr {W}}= (W_1,\dots ,W_N)$ is thrown *N* times, and $\nu _i$ counts how often the outcome *i* occurs. Hence, given the random weights ${\mathscr {W}}$ the offspring numbers $\nu = (\nu _1, \ldots , \nu _N)$ are Multinomial$(N, {\mathscr {W}})$-distributed. Following Kingman’s terminology, we speak of a *paintbox representation* for $\nu$, and call ${\mathscr {W}}$ the underlying (random) *paintbox*.

This construction is iterated over the generations $g\in {\mathbb {Z}}$: Let ${\mathscr {W}}^{(g)}=(W_1^{(g)},\dots ,W_N^{(g)})$ be independent copies of ${\mathscr {W}}$, and denote the individuals in generation *g* by (*i*, *g*), $i \in [N]$. Assume that each individual $(j,g+1)$, $j \in [N] := \{1,\dots ,N\}$ in generation $g+1$, chooses its parent in generation *g*, with conditional distribution

$$\begin{aligned} { {\mathbb {P}}( (i,g) \text { is the parent of } (j,g+1) |{\mathscr {W}}^{(g)})= W_i^{(g)}, \qquad \forall \, i \in [N]. } \end{aligned}$$

where given ${\mathscr {W}}^{(g)}$ the choices of the parents for individuals $\{(j,g+1), j \in [N]\}$ are independent and identically distributed. This results in exchangeable offspring vectors $\nu ^{(g)}$ which are independent and identically distributed over the generations *g*.

For notational simplicity we do not always display dependence of ${\mathscr {W}}^{(g)}$ on the generation *g*, and write ${\mathscr {W}}$ instead. From time to time however we want to emphasise the dependence of ${\mathscr {W}}$ on *N* and therefore write ${\mathscr {W}}^{(N)}$ instead of ${\mathscr {W}}$.

Some exchangeable offspring vectors do not have a paintbox representation, for example a random permutation of the vector $(2,\ldots ,2,0,\ldots ,0)$. Prototypical paintboxes are ${\mathscr {W}} = (\frac{1}{N}, \ldots , \frac{1}{N})$, which leads to the Wright-Fisher model, and the class of Dirichlet$(\alpha ,\ldots ,\alpha )$-distributed random weights. In particular, the offspring distribution with Dirichlet$(1,\ldots ,1)$-distributed paintbox can be seen as a limiting case of the offspring distribution for the model of experimental evolution considered in Baake et al. (2019) and González Casanova et al. (2017).

### A paintbox representation with selection

Let ${\mathscr {W}}^{(g)}$, $g\in {\mathbb {Z}}$, be as in the previous section, and let $s_N \in [0,1)$. Assume each individual carries one of two types, either the *beneficial type* or the *wildtype*. Depending on the type of individual (*i*, *g*) we set

$$\begin{aligned} {\widetilde{W}}_i^{(g)}= (1-s_N)W_i^{(g)} \end{aligned}$$

if (*i*, *g*) is of wildtype and ${\widetilde{W}}_i^{(g)}=W_i^{(g)}$ if (*i*, *g*) is of beneficial type. The probability that an individual is chosen as parent is now given by

4 $$\begin{aligned} {\mathbb {P}}( (i,g) \text { is parent of } (j,g+1) )= \frac{{\widetilde{W}}_i^{(g)}}{\sum _{\ell =1}^{N} {\widetilde{W}}_\ell ^{(g)}} \end{aligned}$$

for all $i,j \in [N]$. Parents are chosen independently for all $i \in [N]$ and the distribution does not change over the generations. If (*i*, *g*) is the parent of $(j,g+1)$ the child $(j,g+1)$ inherits the type of its parent. In particular, this reproduction mechanism leads to offspring numbers that are exchangeable among the beneficial as well as among wildtype individuals.

### The Cannings frequency process

In the previous section we gave a definition for a Cannings model which incorporates selection, by decreasing the random weight of each wildtype individual by the factor $1-s_N$. This allows to define the Cannings frequency process ${\mathcal {X}}=(X_g)_{g\ge 0}$ with state space [*N*] which counts the number of beneficial individuals in each generation *g*.

Assume there are $1 \le k \le N$ beneficial individuals at time *g*; due to the exchangeability of ${\mathscr {W}}^{(g)}$ we may assume that the individuals $(1,g),\ldots , (k,g)$ are the beneficial ones. Given ${\mathscr {W}}^{(g)}={\mathscr {W}}$, the probability that individual $(j,g+1)$ is of beneficial type is then due to (4) equal to

5 $$\begin{aligned} \frac{\sum _{i=1}^{k} W_i}{ \sum _{i=1}^{k} W_i+ {(1-s_N )}\sum _{i=k+1}^{N} W_i}, \end{aligned}$$

and is the same for all $j \in [N]$. Hence, given ${\mathscr {W}}^{(g)}= {\mathscr {W}}$ and given there are *k* beneficial individuals in generation *g*, the number of beneficial individuals in generation $g+1$ has distribution

6 $$\begin{aligned} \text { Bin} \left( N, \frac{\sum _{i=1}^{k} W_i}{ \sum _{i=1}^{k} W_i + {(1-s_N )} \sum _{i=k+1}^{N} W_j} \right) ; \end{aligned}$$

this defines the transition probabilities of the Markov chain ${\mathcal {X}}$.

## Main result

Before we state our main result we specify the assumptions on the paintbox and the strength of selection.

### Definition 1

(*Dirichlet-type weights*) We say that a random vector ${\mathscr {W}}^{(N)}$ with exchangeable components $W_1^{(N)},\ldots , W_N^{(N)}$ is *of Dirichlet-type* if

7 $$\begin{aligned} W_i^{(N)}=\frac{Y_i}{\sum _{\ell =1}^{N} Y_\ell }, \quad i=1,\ldots , N, \end{aligned}$$

where $Y_1, \ldots , Y_N$ are independent copies of a random variable *Y* with ${\mathbb {P}}(Y > 0) =1$.

We assume that

8 $$\begin{aligned} {\mathbb {E}} \left[ \exp (hY) \right] < \infty , \end{aligned}$$

for some $h>0$, which implies the finiteness of all moments of *Y*. The relevance (and possible relaxations) of Condition (8) are discussed further in Remark 2(a), see also the comment in Remark 1(a).

### Remark 1

- The biological motivation for considering Dirichlet-type weights comes from seasonal reproductive schemes. At the beginning of a season a set (of size *N*) of individuals is alive. These individuals and their offspring reproduce and generate a pool of descendants within that season. Only a few individuals from this pool survive till the next season. The number *N* in the model is assumed to be the total number of individuals that make it to the next season. Dirichlet-type weights arise in the asymptotics of an infinitely large pool of offspring; then sampling with and without replacement coincide. Condition (8), which we will require for the proof of Theorem 1 (see also Remark 2), guarantees that the pool of descendants of a single individual is not too large in comparison to the pool of descendants generated by the other individuals. The simplifying assumption $\mathbbm {P}(Y>0)=1$ implies that the weight $W_i^{(N)}$ of a parent cannot be equal to zero. Observe, however, that weights of single parents can be arbitrarily small if (e.g.) *Y* has a density which is continuous and strictly positive in zero.
- The case of a deterministic *Y* corresponds to $W_i^{(N)} \equiv 1/N$, i.e. the classical Wright-Fisher model. If *Y* has a Gamma($\kappa$)-distribution, then ${\mathscr {W}}^{(N)}$ is Dirichlet $(\kappa ,\ldots , \kappa )$-distributed.
  Theorem 1 in Huillet and Möhle (2021), gives a classification of a large class of Cannings models with a paintbox of the form (7) with regard to the convergence of their rescaled genealogies.
- Let $\nu ^{(N)}$ be a sequence of Cannings offspring numbers that are represented by the paintboxes ${\mathscr {W}}^{(N)}$. It is well known (and easily checked) that
  9 $$\begin{aligned} \text {Var}\left( \nu _1^{(N)} \right) = N(N-1){\mathbb {E}}[(W_1^{(N)})^2]. \end{aligned}$$
  If ${\mathscr {W}}^{(N)}$ is of the form (1) with ${\mathbb {E}}[Y^2] < \infty$, which is clearly implied by (8), then (see Huillet and Möhle (2021) Theorem 1 (i)) the right hand side of (9) converges to $\frac{{\mathbb {E}}[Y^2]}{{\mathbb {E}}[Y]^2}$ as $N\rightarrow \infty$.

In view of Remark 1(c) we have for the asymptotic neutral offspring variance

10 $$\begin{aligned} \lim \limits _{N\rightarrow \infty } \text {Var}\left( \nu _1^{(N)} \right) =: \rho ^2= \frac{{\mathbb {E}}[Y^2]}{{\mathbb {E}}[Y]^2}. \end{aligned}$$

Replacing *Y* by $Y' = \frac{Y}{\mathbbm {E}[Y]}$ does not affect (7), hence we can and will assume ${\mathbb {E}}[Y] = 1$ in the proofs, which simplifies (10) to $\rho ^2 = {\mathbb {E}}[Y^2]$ (and makes (10) consistent with the notation of Huillet and Möhle (2021)). Under the assumption ${\mathbb {E}}[Y^4] < \infty$, which is implied by (8) as well, the following asymptotics is valid

11 $$\begin{aligned} \text {Var}\left( \nu _1^{(N)} \right) =\rho ^2 +O(N^{-1}), \text{ as } N\rightarrow \infty . \end{aligned}$$

We will discuss the relevance of this asymptotics in Remark 2(b), and prove it in Lemma 2(b).

Turning to the selective advantage, we assume that for a fixed $\eta \in (0,\frac{1}{4})$ the sequence $(s_N)$ obeys

12 $$\begin{aligned} N^{-\frac{1}{2} +\eta } \le s_N \le N^{-\eta } , \end{aligned}$$

which we call the regime of *moderately strong selection*, thus generalizing the corresponding notion introduced in Sect. 1. (Note that (12) has an analogue in the regime of *moderately weak selection* as discussed in Boenkost et al. (2021)). In order to connect to (2) we define

13 $$\begin{aligned} b_N := -\frac{ \ln s_N}{\ln N} \end{aligned}$$

which is equivalent to $s_N = N^{-b_N}$, with (12) translating to

$$\begin{aligned} \eta \le b_N \le \frac{1}{2} - \eta . \end{aligned}$$

We now state our main result on the asymptotics (as $N\rightarrow \infty$) of the fixation probability of the Cannings frequency process $(X_g^{(N)}) = (X_g)$ defined in Sect. 2.3. Note that the Markov chain $(X_g)$ has the two absorbing states 0 and *N*, with the hitting time of $\{0,N\}$ being a.s. finite for all *N*.

### Theorem 1

(Haldane’s formula) Assume that Conditions (7), (8) and (12) are fulfilled. Let $(X_g)_{g \ge 0}$ be the number of beneficial individuals in generation *g*, with $X_0= 1$. Let $\tau =\inf \left\{ g \ge 0 : X_g \in \left\{ 0,N\right\} \right\}$, then

14 $$\begin{aligned} {\mathbb {P}}(X_{\tau }=N) { \sim \frac{2 s_N}{\rho ^2},\qquad \text { as } N \rightarrow \infty .} \end{aligned}$$

We give the proof of Theorem 1 in Sect. 5, after preparing some auxiliary results in Sect. 4. Next we give a strategy of the proof and its main ideas, with an emphasis on the role of Condition (12). In Remark 2 we discuss possible relaxations of Condition (8) and the boundary case $b=\frac{1}{2}.$

The proof of Theorem 1 is divided into three parts, corresponding to three growth phases of ${\mathcal {X}}$. Concerning the *first phase* we show that the probability to reach the level $N^{b+\delta }$ is $\frac{2 s_N}{\rho ^2}(1+o(1))$, for some small $\delta >0$ and $b:=b_N$; this is the content of Proposition 1. The proof is based on stochastic domination from above and below by slightly supercritical Galton–Watson processes $\overline{ {\mathcal {Z}} }$ and $\underline{ {\mathcal {Z}} }$ with respective offspring distributions (29) and (30).

To construct a Galton–Watson stochastic upper bound $\overline{ {\mathcal {Z}} }$ of ${\mathcal {X}}$ in its initial phase, we recall that the transition probabilities of ${\mathcal {X}}$ are mixed Binomial specified by (6). Using (7) we approximate (5) from above by

15 $$\begin{aligned} \frac{ 1+s_N +o(s_N) }{N }\sum _{\ell =1}^{k} Y_\ell . \end{aligned}$$

As we will show in Lemma 4, this is possible with probability $1-O(\exp (-c'N^{1-2\alpha }))$ for some $\alpha < \frac{1}{2}$ and $c'>0$, and for $k \le N^{b+\delta }$ with $b+\delta < 1/2$. We will then be able to dominate the mixed Binomial distribution (6) by the mixed Poisson distribution with random parameter (15), again up to an error term of order $o(s_N)$. Noting that (15) is a sum of independent random variables, we arrive at the upper Galton–Watson approximation for a single generation. For any small $\varepsilon >0$ this can be repeated for $N^{b+\varepsilon }$ generations, which (as an application of Lemma 1 will show) is enough to reach either the level 0 or the level $N^{b+\delta }$ with probability $1-o(s_N)$.

To obtain a Galton–Watson stochastic lower bound $\underline{ {\mathcal {Z}} }$ of ${\mathcal {X}}$ in its initial phase, we adapt an approach that was used in González Casanova et al. (2017) in a related situation. As in Sect. 2.1, number the individuals in generation *g* by (*i*, *g*), now with $(1,g), \ldots , (X_g,g)$, being the beneficial individuals, and denote by $\omega _i^{(g)}$ the number of children of the individual (*i*, *g*), $1\le i \le X_g$. As will be explained in the proof of Lemma 5, as long as $X_g$ has not reached the level $N^{b+\delta }$, the distribution of $\omega _i^{(g)}$ can be bounded from below by a mixed binomial distribution

$$\begin{aligned} \text { Bin} \left( N - \lceil N^{b+\delta } \rceil , Y_1 \frac{1+s_N +o(s_N)}{N}\right) \end{aligned}$$

with probability $1-O(\exp (-N^{\varepsilon }))$ for some sufficiently small $\varepsilon >0$, again for $b + \delta <1/2$. A suitable stopping and truncation at the level $N^{b+\delta }$ will give the Galton–Watson process approximation from below for the first phase.

We will verify in Sect. 4.1 that both slightly supercritical branching processes $\underline{{\mathcal {Z}}}$ and $\overline{ {\mathcal {Z}} }$ reach the level $N^{b+\delta }$ with probability $\frac{2s_N}{\rho ^2} (1+o(1))$.

As to the *second phase*, we will argue in Sect. 5.2 that, after reaching the level $N^{b+\delta }$ the Cannings frequency process ${\mathcal {X}}$ will grow to a macroscopic fraction $\varepsilon N$ with high probability. If the frequency of beneficial individuals is at least $N^{b+\delta }$ (but still below $\varepsilon N$), then in a single generation the frequency of beneficial individuals grows in expectation at least by $1 + (1- \varepsilon ) s_N + o(s_N)$. Hence, $c s_N ^{-1} \ln N$ generations after ${\mathcal {X}}$ has reached the level $N^{b+\delta }$, the expected value of the process ${\mathcal {X}}$ reaches the level $2 \varepsilon N$. Similarly one bounds the variance produced in a single generation and derives from this an estimate for the variance accumulated over $c s_N ^{-1} \ln N$ generations. This bound being sufficiently small, an application of Chebyshev’s inequality yields that (after $c s_N ^{-1} \ln N$ generations) ${\mathcal {X}}$ crosses the level $\varepsilon N$ with probability tending to 1 after reaching the level $N^{b+\delta }$.

In Sect. 5.3) we deal with the *last phase*, and will show that the fixation probability tends to 1 as $N \rightarrow \infty$ if we start with at least $\varepsilon N$ individuals of beneficial type. Here we use the representation for the fixation probability that is based on a sampling duality between the Cannings frequency process and the Cannings ancestral selection process (CASP) which was provided in Boenkost et al. (2021). For a subregime of moderately weak selection the claim will follow quickly from the representation formula combined with a concentration result for the equilibrium distribution of the CASP that was proved in Boenkost et al. (2021). To complete the proof we will then argue that both the CASP and the representation of the fixation probability depend on the selection parameter in a monotone way.

### Remark 2

- With some additional work the assumption (8) of the existence of some exponential moment of *Y* can be relaxed to some weaker moment condition. In order not to overload the present paper, we restrict here to a sketch.
  In Lemma 5 we couple the frequency process of the beneficial individuals with Galton–Watson processes for $N^{b+\delta }$ generations. By means of the estimates in Lemma 3 and Lemma 4 we show that these couplings hold for a single generation with probability $1-O(\exp (-N^{c'}))$ for some appropriate $c'>0$. Since we need the couplings to hold for $N^{b+\delta }$ generations, it suffices that the couplings hold in a single generation with probability $1 - O(N^{-2(b + \delta )})$ for some $\delta >0$ (since in this case the probability of the coupling to fail is $o(s_N)$ and therefore can be neglected with regard to (14)). Such probability bounds can also be obtained under weaker assumptions on the distribution of the random variable *Y*. Assume e.g. that *Y* has a regularly varying tail, i.e. $\mathbbm {P} (Y >x)\sim x^{-\beta } L(x)$ for some $\beta >0$ and *L* is a slowly varying function. For the proof of Lemma 5 we need to estimate the probability of the event figuring in Lemma 3 with $b<c \le 1$ and the probability of the event figuring in Lemma 4 with $b<\alpha <\frac{1}{2}$. To show that these probabilities are of order $O(N^{-2(b + \delta )})$ we only need that $\mathbbm {P}\left( \sum _{i=1}^n Y_i > x \right) = O(n^{-2(b + \delta )})$ (since the remaining probability in Lemma 3 can be estimated with Hoeffding’s inequality, see Hoeffding (1963)) with $n = N, x = N^{1-\alpha }$ in Lemma 4 and $n= N^c, x= N^{c}$ in Lemma 3. The asymptotics (3.2) in Mikosch and Nagaev (1998) states that $\mathbbm {P}\left( \sum _{i=1}^n Y_i > x \right) \sim n x^{-\beta } L(x)$. Consequently, we need to choose $\beta >0$ such that $N^{1 - \beta (1-\alpha )} L(N^{1-\alpha }) = O(N^{-2(b + \delta )})$ as well as $N^{c - \beta c} L(N^c) = O(N^{-2(b + \delta )})$. This works for all choices of $0< b< \tfrac{1}{2}$, provided that $\beta \ge 4$.
  It would be nice to have a proof of the asymptotics (14) under the assumption that the 4th moment of *Y* is finite, even without the assumption of a regularly varying tail.
  The investigation of the analogue to (14) in the absence of finite second moments, i.e. for Cannings models with heavy-tailed offspring distributions, is the subject of ongoing research, and will be treated in a forthcoming paper.
- Relation (11) will be used in the proof of Lemma 6. Moreover, this relation is also instrumental in the companion paper Boenkost et al. (2021) (on the regime of moderately weak selection). The special case $n=3$ in Lemma 2 a) shows that the assumption $\mathbb E[Y^3] <\infty$ implies
  $$\begin{aligned} { {\mathbb {E}}[(W_1^{(N)})^3] = O(N^{-3})}. \end{aligned}$$
  This gives a rate of decay $O(N^{-2})$ for the triple coalescence probability (and is the moment condition (3.6) in Boenkost et al. (2021)).
  Condition (8) (on the existence of an exponential moment of *Y*) guarantees the Haldane asymptotics (14) for Cannings models with weights of Dirichlet type also in the whole regime of moderately weak selection $N^{-1 +\eta } \le s_N \le N^{-\frac{1}{2} -\eta }$ without any further assumption. In particular the assumption on the finiteness of a negative moment of *Y* in Boenkost et al. (2021), Lemma 3.7 b), is unnecessary. Indeed, in the proof of Lemma 2(a) we show that $\mathbbm {E}[(W_1^{(N)})^n] \le \left( \frac{2}{N}\right) ^n( \mathbbm {E}[Y^n] + o(1))$. As shown in the proof of Lemma 3.7(b) in Boenkost et al. (2021) Condition (8) guarantees that for a sequence $(h_N)$ with $h_N \rightarrow \infty$ and $h_N \in O(\log N)$ for all $n\le 2 h_N$ we can estimate $\mathbbm {E}[Y^n]$ from above by $C \left( \frac{2h_N}{c}\right) ^n$ for appropriate constants $C,c>0$. Consequently, for *N* sufficiently large we have $\mathbbm {E}[(W_1^{(N)})^n] \le \left( \frac{Kh_N}{N} \right) ^n$ for some appropriate constant $K>0$, that is Condition (3.8) in Boenkost et al. (2021) is fulfilled.
- It seems a mathematically intriguing question whether in the regime of moderate selection all Cannings models which admit a paintbox representation with Dirichlet-type weights and are in Kingman domain of attraction, also follow the Haldane asymptotics (14).
  An example of a sequence of Cannings models (with weights *not* of Dirichlet-type) which fulfil Möhle’s condition but do not follow the Haldane asymptotics, is the following. In each generation a randomly chosen individual gets weight $N^{-\gamma }$, $0<\gamma <\frac{1}{2}$ and all the other individuals have a weight of $\frac{1-N^{-\gamma }}{N-1}$. Then we have ${\mathbb {E}} \left[ W_1^2 \right] \sim N^{-1-2\gamma }$ and ${\mathbb {E}} \left[ W_1^3 \right] =o({\mathbb {E}} \left[ W_1^2 \right] )$, therefore by Möhle’s criterion the genealogy lies in the attraction of Kingman’s coalescent. However, the Haldane asymptotics would predict that the survival probability is of order $s_N/(N^2 N^{-1-2\gamma })\sim N^{-1-b+2\gamma }$, which for $\gamma < b/2$ is $\ll N^{-1}$. Since the fixation probability of a beneficial allele cannot be smaller than the fixation probability under neutrality (which is $\frac{1}{N}$), (14) must be violated in this example.
- The present work together with the approach in Boenkost et al. (2021) does not cover the boundary case $b=\frac{1}{2}$. A quick argument why our arguments cannot be extended simply to the boundary case is the following. We show that once the beneficial type exceeds (in the order of magnitude) the frequency $s_N^{-1}= N^b$ it goes to fixation with high probability. In the regime $b<\frac{1}{2}$ we use couplings with Galton–Watson processes to show that this threshold is reached with probability $\frac{2s_N}{\rho ^2}(1+ o(1))$. However, these couplings are not guaranteed as soon as collisions occur, i.e. when beneficial individuals are replacing beneficial individuals. By the well known ”birthday problem“ collisions are common as soon as $N^{\frac{1}{2}}$ individuals are of the beneficial type. Therefore we require $N^{b} \ll N^{\frac{1}{2}}$, i.e. $b <\tfrac{1}{2}$.
  In the light of the results of the present paper and of Boenkost et al. (2021), there is little reason to doubt that the assertion of Theorem 1 should fail in the boundary case $b=1/2$. However, the question remains open (and intriguing) whether then the backward or the forward approach (or a combination of both) is the appropriate tool for the proof.

## Auxiliary results

### Slightly supercritical Galton–Watson processes

Throughout this subsection, $(s_N)_{N\in \mathbbm {N}}$ is a sequence of positive numbers converging to 0, $\sigma ^2$ is a fixed positive number, and $Z^{(N)}=(Z_n^{(N)})_{n\ge 0}$, $N=1, 2, \ldots$ are Galton–Watson processes with offspring expectation

16 $$\begin{aligned} {\mathbb {E}}_1[Z_1^{(N)}] = 1+s_N+o(s_N), \end{aligned}$$

offspring variance $\sigma ^2 +o(1)$ and uniformly bounded third moments ${\mathbb {E}}_1[(Z_1^{(N)})^3]$. Unless stated otherwise we assume that $Z_0^{(N)}=1$. We write

17 $$\begin{aligned} \phi _N := {\mathbb {P}}\left( \lim _{n\rightarrow \infty } Z_n^{(N)}=\infty \right) = 1-{\mathbb {P}}\left( Z_n^{(N)} = 0 \text{ for } \text{ some } n > 1\right) \end{aligned}$$

for the survival probability of $(Z^{(N)})$ and observe

18 $$\begin{aligned} \phi _N{\sim } \frac{2 s_N}{\sigma ^2}. \end{aligned}$$

The derivation and discussion of the asymptotics (18) has a venerable history, a few key references being Haldane (1927), Kolmogorov (1938), Eshel (1981), Hoppe (1992), Athreya (1992) Theorem 3, Haccou et al. (2005) Theorem 5.5.

Lemma B.3 in González Casanova et al. (2017) gives a statement on the asymptotic probability that $Z^{(N)}$ either quickly dies out or reaches a certain (moderately) large threshold. The following lemma improves on this in a twofold way. It dispenses with the assumption $s_N \sim c N^{-b}$ for a fixed $b\in (0,1)$ and more substantially, it gives a *quantitative* estimate for the probability that, given non-extinction, the (moderately) large threshold is reached quickly.

#### Lemma 1

Fix $\delta >0$, and let $T^{(N)}:=\inf \{n\ge 0 : Z_n^{(N)} \notin \{1,2,\ldots ,\lceil (\frac{1}{s_N} )^{1+\delta } \rceil \}$. Then, for all $\varepsilon >0$

19 $$\begin{aligned} {\mathbb {P}}_1\left( T^{(N)}> (1/s_N)^{(1+\varepsilon )} \right) = O\left( \exp \left( -cs_N^{- \varepsilon /2} \right) \right) , \end{aligned}$$

with $$c = - \log \left( \tfrac{7}{8}\right) $$.

#### Proof

Observe that

20 $$\begin{aligned} {\mathbb {P}}_1\left( T^{(N)}> \left( 1/s_N\right) ^{1+\varepsilon } \right)&\le {\mathbb {P}}_1\left( T^{(N)}> (1/s_N)^{1+\varepsilon } \Big | Z^{N} \text { survives} \right) \nonumber \\&\qquad +{\mathbb {P}}_1\left( T^{(N)}> (1/s_N)^{1+\varepsilon }\Big | Z^{N} \text { dies out} \right) . \end{aligned}$$

In Part 1 of the proof we will estimate the first probability on the r.h.s. of (20); this will give the above-mentioned improvement of Lemma B.3 in González Casanova et al. (2017). Part 2 of the proof deals with the second probability on the r.h.s. of (20).

*Part 1. * Like in the proof of Lemma B.3 in González Casanova et al. (2017) we obtain an upper bound on the time at which the process $Z^{(N)}$ reaches the level $(1/s_N)^{1+\delta }$ given survival, by considering the process $Z^{\star }=(Z_n^{\star })_{n\ge 0}$ consisting of the immortal lines of $Z^{(N)}$ conditioned to non-extinction. (For simplicity of notation we drop a superscript *N* in $Z^{\star }$.) Let $\phi _N$ denote the survival probability of $Z^{(N)}$ as in (17). The offspring distribution of $Z^{\star }$ arises from that of $Z^{(N)}$ as

21 $$\begin{aligned} {\mathbb {P}}_1(Z_1^{\star } = k) = \frac{1}{\phi _N^{}} \mathbb E_1\left[ {{Z_1^{(N)}}\atopwithdelims (){k}}\phi _N^k (1-\phi _N)^{Z_1^{(N)}-k}\right] , \quad k \ge 1. \end{aligned}$$

(see Lyons and Peres 2017 Proposition 5.28). In particular one has

$$\begin{aligned} {\mathbb {E}}_1[Z_1^{\star }] = \frac{1}{\phi _N} {\mathbb {E}}_1[Z_1^{(N)}\phi _N ] = {\mathbb {E}}_1[ Z_1^{(N)}]. \end{aligned}$$

Denote, as usual, for a random variable *X* and an event *A* by ${\mathbb {E}} \left[ X;A \right] :={\mathbb {E}} \left[ X \mathbbm {1}_A \right]$. Furthermore,

$$\begin{aligned} {\mathbb {E}}_1[Z_1^{\star }; Z_1^{\star }\ge 3] \le \frac{1}{\phi _N^{}} {\mathbb {E}}_1\left[ {{Z_1^{(N)}}\atopwithdelims (){3}}\phi _N^3\right] = O(\phi _N^2) \end{aligned}$$

because of the assumed uniform boundedness of the third moments of $Z_1^{(N)}$. These two relations together with (16), (18) and the fact that $\mathbb E_1[Z_1^{\star }; Z_1^{\star }= 1]\le 1$ immediately give a lower bound for ${\mathbb {E}}_1[Z_1^{\star }; Z_1^{\star }= 2]\le 1$, implying that for any $\beta \in (0,1)$

22 $$\begin{aligned} {\mathbb {P}}_1 ( Z_1^{\star } \ge 2) \ge \beta s_N , \qquad {\mathbb {P}}_1(Z_1^{\star } =1) \le 1-\beta s_N. \end{aligned}$$

Hence the process $Z^{(N)}$, when conditioned on survival, is bounded from below by the counting process $Z^{\star }$ of immortal lines, which in turn is bounded from below by the process ${\widetilde{Z}}=({\widetilde{Z}}_n ) _{n \ge 0 }$ with offspring distribution

$$\begin{aligned} \nu = (1-\beta s_N) \delta _1 + \beta s_N \delta _2. \end{aligned}$$

So far we closely followed the proof in González Casanova et al. (2017), but now we deviate from that proof to obtain the rate of convergence claimed in (19).

An upper bound for the time ${\widetilde{T}}:= \inf \{n \ge 0 : {\widetilde{Z}}_n \ge (1/s_N)^{1+\delta } \}$ also gives an upper bound for the time $T^{(N)}$. The idea is now to divide an initial piece of $k \le (1/s_N)^{(1+\varepsilon )}$ generations into $\lfloor {(1/s_N)^{\varepsilon /2}} \rfloor$ parts, each of $n_0 \le (1/s_N)^{(1+\varepsilon /2)}$ generations. Because of the immortality of ${\widetilde{Z}}$ and the independence between these parts we obtain immediately that

$$\begin{aligned} {\mathbb {P}}({{\widetilde{T}}} \ge (1/s_N)^{(1+\varepsilon )})&\le {\mathbb {P}}_1({\widetilde{Z}}_{ j } \le (1/s_N)^{1+\delta } \text{ for } j=1,\ldots , k )\\&\le \left( \mathbb P_1({\widetilde{Z}}_{n_0}\le (1/s_N)^{1+\delta } )\right) ^{\lfloor {(1/s_N)^{\varepsilon /2}} \rfloor } \end{aligned}$$

We then bound ${\mathbb {P}}_1 ( {\widetilde{Z}}_{n_0} >(1/s_N)^{1+\delta } )$ from below by an application of the Paley-Zygmund inequality in its form

23 $$\begin{aligned} {\mathbb {P}}\left( X\ge \frac{{\mathbb {E}}[X]}{2}\right) \ge \frac{1}{4} \frac{({\mathbb {E}}[X])^2}{{\mathbb {E}}[X^2]}, \end{aligned}$$

where *X* is a non-negative random variable (with finite second moment). For a supercritical Galton–Watson process with offspring expectation *m* and offspring variance $\sigma ^2$ the *n*-th generation offspring expectation and *n*-th generation offspring variance $\sigma ^2_n$ are given by $m^n$ and $\sigma ^2 m^n (m^n -1)/(m^2 -m)$ (see Athreya and Ney (1972), p.4). Hence, we obtain

24 $$\begin{aligned} {\mathbb {E}}_1[{\tilde{Z}}_n]&= (1+ \beta s_N)^n , \nonumber \\ \mathrm{Var}_1[{\tilde{Z}}_n]&= \frac{\beta s_N (1-\beta s_N) (1+\beta s_N)^n ((1+ \beta s_N )^n -1) }{(1+\beta s_N)^2 -(1+\beta s_N)}. \end{aligned}$$

We choose the smallest $n_0$ such that

25 $$\begin{aligned} {\mathbb {E}}_1{ [{\widetilde{Z}}_{n_0}] } { \ge } 2 (1/s_N)^{1+\delta }. \end{aligned}$$

Observe that $n_0 \sim \frac{1}{\beta s_N} \log (2 (\frac{1}{s_N})^{1+\delta } )$ which ensures that $(1/s_N)^{\varepsilon /2} n_0 \le (1/s_N)^{1+\varepsilon }$ for *N* large enough. We now estimate ${\mathbb {E}}_1{ [({\widetilde{Z}}_{n_0})^2] }$ using (24) as follows

$$\begin{aligned} {\mathbb {E}} \left[ {\widetilde{Z}}_{n_0}^2 \right]&= \frac{\beta s_N (1-\beta s_N) (1+\beta s_N)^{n_0} ((1+ \beta s_N )^{n_0} -1) }{(1+\beta s_N)^2 -(1+\beta s_N)} + (1+\beta s_N)^{2n_0} \\&\le \frac{\beta s_N (1+\beta s_N)^{2n_0} }{ \beta s_N + (\beta s_N )^2 } + (1+\beta s_N)^{2n_0} \le 2 (1+\beta s_N)^{2n_0}. \end{aligned}$$

Applying (23) with $X:= {{\widetilde{Z}}}_{n_0}$ yields

$$\begin{aligned} {\mathbb {P}}_1 \left( {\widetilde{Z}}_{n_0} \ge \frac{1}{2} {\mathbb {E}} \left[ {\widetilde{Z}}_{n_0} \right] \right) \ge \frac{1}{4} \frac{(1+\beta s_N)^{2n_0} }{2 (1+\beta s_N)^{2n_0}}= \frac{1}{8}, \end{aligned}$$

which because of (25) implies ${\mathbb {P}}_1( {\widetilde{Z}}_{n_0} \le (1/s_N)^{1+\delta } ) \le \frac{7}{8}$. If after time $n_0$ the process ${\widetilde{Z}}_{n_0}$ is still smaller than our desired bound $(1/s_N)^{1+\delta }$, we can iterate this argument $\lfloor {(1/s_N)^{\varepsilon /2}} \rfloor$ times and arrive at

$$\begin{aligned} ({\mathbb {P}}_1( {\widetilde{Z}}_{n_0} \le (1/s_N)^{1+\delta } )^{\lfloor {(1/s_N)^{\varepsilon /2}} \rfloor } \le \left( \frac{7}{8}\right) ^{\lfloor {(1/s_N)^{\varepsilon /2}} \rfloor } = \exp (-c \lfloor {(1/s_N)^{\varepsilon /2} } \rfloor ), \end{aligned}$$

with $c = -\log \frac{7}{8}$. This gives the desired bound for the first term in (20).

*Part 2.* We now turn to the second term on the r.h.s. of (20). Define

$$\begin{aligned}T_0^{(N)}:= \inf \{ n \ge 0 : Z^{(N)}_n=0\}. \end{aligned}$$

Obviously $T^{(N)}\le T_0^{(N)}$, and so it suffices to prove

26 $$\begin{aligned} {\mathbb {P}}\left( T_0^{(N)}> (1/s_N)^{1+\varepsilon }|Z^{(N)} \text { dies out}\right) \le \exp (-\beta s_N^{-\varepsilon } (1+o(1))). \end{aligned}$$

This proof follows closely that of the second part of Lemma B.3 in González Casanova et al. (2017); we include it here for completeness.

We observe

$$\begin{aligned} {\mathbb {E}}_1\left[ Z_1^{(N)}| Z^{(N)} \text { dies out} \right]&= \frac{1}{1-\phi _N} {\mathbb {E}}_1\left[ (1-\phi _N)^{Z_1^{(N)}} Z_1^{(N)}\right] \\&= {\mathbb {E}}_1\left[ (1-\phi _N)^{Z_1^{(N)}-1} Z_1^{(N)}\right] = {\mathbb {P}}_1(Z_1^{\star } =1), \end{aligned}$$

where the first and the last equality follow from the branching property and from (21), respectively. We have shown in (22) that ${\mathbb {P}}_1(Z_1^{\star } =1) \le 1- \beta s_N +o(s_N)$ and hence we can conclude

$$\begin{aligned} {\mathbb {E}} \left[ Z_{\lfloor { (1/s_N)^{1+\varepsilon }} \rfloor }|Z^{(N)} \text { dies out } \right]&\le (1-\beta s_N +o(s_N))^{\lfloor {(1/s_N)^{1+\varepsilon }} \rfloor }\\&\le \exp (-\beta s_N^{-\varepsilon } (1+o(1))). \end{aligned}$$

Finally, an application of Markov’s inequality yields (26)

$$\begin{aligned} {\mathbb {P}}(T_0^{(N)}> (1/s_N)^{1+\varepsilon }|Z^{(N)} \text { dies out })&\le {\mathbb {P}}(Z_{\lfloor {(1/s_N)^{1+\varepsilon }} \rfloor } \ge 1|Z^{(N)} \text { dies out })\\&\le \exp (-\beta s_N^{-\varepsilon } (1+o(1))). \end{aligned}$$

$$\square $$

### Estimates on the paintbox

The following lemma provides the asymptotics (11) as well as the moment bounds for the Dirichlet-type weights that were addressed in Remark 2(b).

#### Lemma 2

(Moments of the weights) Let $Y, Y_1, \ldots , Y_N$ be iid positive random variables with $\mathbbm {E}[Y]=1$ and $\rho ^2 = \mathbbm {E}[Y^2].$ We abbreviate $H_N:=Y_1+\cdots +Y_N$.

(a) Assume $\mathbbm {E}[Y^n] < \infty$ for some $n\in \mathbbm {N}$. Then

$$\begin{aligned} { \mathbbm {E}\left[ \left( \frac{Y}{(Y +H_N)}\right) ^n \right] = O(N^{-n}).} \end{aligned}$$

(b) Assume $${\mathbb {E}}[Y^4] < \infty $$. Then

$$\begin{aligned} {\mathbbm {E}\left[ \left( \frac{Y}{(Y +H_N)}\right) ^2 \right] = \frac{\rho ^2}{N^2} + O(N^{-3}).} \end{aligned}$$

#### Proof

a) Consider the event $F_N:= \{\frac{H_N}{N} \le \frac{1}{2}\}$. First we note that

$$\begin{aligned} {\mathbb {E}} \left[ \left( \frac{Y}{(Y +H_N)}\right) ^n \right]&\le {\mathbb {E}} \left[ \left( \frac{Y}{Y+H_N}\right) ^n\mathbbm {1}_{F_N} \right] +{\mathbb {E}} \left[ \left( \frac{Y}{H_N}\right) ^n\mathbbm {1}_{F_N^c} \right] \\&\le {\mathbb {P}} \left( F_N \right) + \left( \frac{2}{N}\right) ^n {\mathbb {E}} \left[ Y^n \right] . \end{aligned}$$

Let *K* be so large that ${\mathbb {E}}[Y\wedge K] > \frac{1}{2}$. Hoeffding’s inequality applied to the sample mean of i.i.d. copies of the bounded random variable $Y \wedge K$ implies that $\mathbb P(F_N)$ decays exponentially fast. Since ${\mathbb {E}} \left[ Y^n \right]$ is bounded this yields the claim.

(b) We observe that

$$\begin{aligned} \mathbbm {E}\left[ \frac{Y^2}{(Y + H_N)^2} \right]&= \frac{1}{N^2} \mathbbm {E}\left[ \frac{Y^2}{\left( \frac{Y}{N} +\frac{H_N}{N} \right) ^2 } \right] . \end{aligned}$$

Let $E^{(1)}_N: = \{\frac{H_N }{N} > \frac{5}{4}\}, \, E^{(2)}_N := \{ \frac{H_N}{N} < \frac{3}{4} \}$ and $E^{(3)}_N := \{Y > \frac{N }{4}\}.$ Markov’s inequality applied to $\mathbbm {P}\left( \left( \sum _{i=1}^{N} (Y_i-1) \right) ^4 \ge \frac{N^4}{4^4}\right)$ together with the assumption ${\mathbb {E}}[Y^4] < \infty$ implies $\mathbbm {P}(E_N^{(1)}) = O(N^{-2})$. Likewise, $\mathbbm {P}(E_N^{(3)}) = O(N^{-4})$. Furthermore, let *K* be so large that ${\mathbb {E}}[Y \wedge K] > \tfrac{3}{4}$. Again, Hoeffding’s inequality applied to the sample mean of i.i.d.copies of the bounded random variable $Y \wedge K$ together with monotonicity imply that ${\mathbb {P}}(E_N^{(2)})$ decays exponentially; a fortiori we have ${\mathbb {P}}(E_N^{(2)})= O(N^{-3})$.

Let $E_N:= E_N^{(1)} \cup E_N^{(2)} \cup E_N^{(3)}$. We have $\mathbbm {E}\left[ \frac{Y^2}{\left( \frac{H_N}{N} + \frac{Y}{N}\right) ^2} \mathbbm {1}_{E_N} \right] = O(N^{-1})$ since on $E_N^{(1)}$ we have $\frac{Y^2}{\left( \frac{H_N}{N} + \frac{Y}{N} \right) ^2} \le Y^2$ and on $E_N^{(2)}$ and $E_N^{(3)}$ we have $\frac{Y^2}{\left( \frac{H_N}{N} + \frac{Y}{N}\right) ^2} \le N^2$. Hence, it remains to show that $\mathbbm {E}\left[ \frac{Y^2}{\left( \frac{H_N}{N} + \frac{Y}{N}\right) ^2} \mathbbm {1}_{E_N^c} \right] = \rho ^2 + O(N^{-1}).$ Define $Z_N := \frac{1}{\sqrt{N}} (H_N -N)$ and observe

$$\begin{aligned} \mathbbm {E}\left[ \frac{Y^2}{\left( \frac{H_N}{N} + \frac{Y}{N}\right) ^2 } \mathbbm {1}_{E_N^c} \right] = \mathbbm {E}\left[ \frac{Y^2}{\left( 1 + \frac{Z_N}{ \sqrt{N}} + \frac{Y}{ N}\right) ^2 } \mathbbm {1}_{E_N^c} \right] . \end{aligned}$$

Abbreviate $R_N = 2 \left( \frac{Z_N}{ \sqrt{N}} + \frac{Y}{N}\right) +\left( \frac{Z_N}{ \sqrt{N}} + \frac{Y}{N}\right) ^2$. On $E_N^c$ we have $-\frac{1}{2} \le R_N$ and hence

$$\begin{aligned} 1 - R_N \le \frac{1}{\left( \frac{H_N}{N} + \frac{Y}{N}\right) ^2}= \frac{1}{1 + R_N} \le 1 - R_N + 2 R_N^2. \end{aligned}$$

Thus

27 $$\begin{aligned} \mathbbm {E}[ Y^2 (1- R_N) \mathbbm {1}_{E_N^c}]&\le \mathbbm {E}\left[ \frac{Y^2}{\left( \frac{H_N-N}{N} + \frac{Y}{N}\right) ^2} \mathbbm {1}_{E_N^c} \right] \nonumber \\&\le \mathbbm {E}[Y^2 (1- R_N + 2 R_N^2) \mathbbm {1}_{E_N^c}]. \end{aligned}$$

By Cauchy-Schwarz we have $\mathbbm {E}[Y^2 \mathbbm {1}_{E_N^c}] = \mathbbm {E}[Y^2] + O(N^{-1})$. Similarly, $\mathbbm {E}[Y^2 Z_N \mathbbm {1}_{E_N}] = \mathbbm {E}[Y^2 Z_N] + O(N^{-1}) = O(N^{-1})$, since $\mathbbm {E}[Y^2 Z_N]$ vanishes due to the independence of *Y* and $Z_N$. The remaining terms in (27) are $O(N^{-1})$ as well, which completes the proof of Lemma 2. $\square$

We now prove a bound on the deviations for the total weight of *k* individuals.

#### Lemma 3

(Large deviations bound for a moderate number of random weights) Let $(Y_i)$ and $(W^{(N)}_i)$ satisfy (7), (8), $\mathbbm {E}[Y]=1$ and let $k = k_N \le N^{c}$ for some $0<c \le 1$. Then for all $\varepsilon > 0$ there exists a positive constant $c_\varepsilon$ depending only on $\varepsilon$ and the distribution of *Y* such that

28 $$\begin{aligned} {\mathbb {P}} \left( \sum _{i=1}^{k} W_i^{(N)} \ge (1+\varepsilon ) N^{c-1} \right) = O( \exp (-c_{\varepsilon } N^{c} ) ). \end{aligned}$$

#### Proof

This follows by a combination of two Cramér bounds. Indeed, the l.h.s. of (28) is by assumption bounded from above by

$$\begin{aligned} {\mathbb {P}} \left( \frac{\sum _{i=1}^{\lceil N^c \rceil } Y_i}{\sum _{j=1}^{N}Y_j} \ge (1+\varepsilon ) N^{c-1} \right) . \end{aligned}$$

Abbreviating $E:=\{ \sum _{j=1}^{N} Y_j \ge (1-\varepsilon ') N\}$ with $\varepsilon '$ such that $(1+\varepsilon )(1-\varepsilon ')>1$ we estimate the latter probability from above by

$$\begin{aligned}&{\mathbb {P}} \left( \sum _{i=1}^{\lceil N^c \rceil } Y_i \ge (1+\varepsilon ) N^{c-1} \sum _{j=1}^{N} Y_j , E \right) +{\mathbb {P}} \left( E^c \right) \\&\quad { \le {\mathbb {P}} \left( \sum _{i=1}^{\lceil N^c \rceil } Y_i \ge N^c (1+\varepsilon )(1-\varepsilon ') \right) +{\mathbb {P}} \left( E^c \right) }\\&\quad { = O(e^{-N^c I((1+\varepsilon )(1-\varepsilon ') )} ) +O(e^{-N I(1-\varepsilon ' )} ) }, \end{aligned}$$

denoting by *I*(*y*) the rate function of *Y*. Due to (8) *I*(*y*) exists around ${\mathbb {E}}[Y]=1$ and is strictly positive for $y \ne 1$ (see Dembo and Zeitouni (1994) Theorem 2.2.3). This yields an upper bound of $O(\exp (-c_\varepsilon N^c))$ with $c_\varepsilon =\min \{ I((1+\varepsilon )(1-\varepsilon ')), I(1-\varepsilon ') \}$. $\square$

The next lemma gives stochastic upper and lower bounds for the sums of the random weights in terms of sums of the independent random variables $Y_i$.

#### Lemma 4

(Bounds for the random weights) Assume that Conditions (7) and (8) are fulfilled and $\mathbbm {E}[Y]=1$. Let $0<\alpha <\frac{1}{2}$, then for $k=k_N \le N$

$$\begin{aligned}&{\mathbb {P}} \left( \frac{1- N^{-\alpha } }{N } \sum _{i=1}^{k} Y_i \le \sum _{i=1}^k W_i^{(N)} \le \frac{1+ N^{-\alpha } }{N } \sum _{i=1}^{k} Y_i \right) \\&\quad \ge 1- \exp \left( - c' N^{1-2\alpha }\right) (1+o(1)), \end{aligned}$$

for some $$c'>0$$.

#### Proof

It suffices to show

$$\begin{aligned} {\mathbb {P}} \left( \left| \frac{N }{\sum _{j=1}^{N} Y_j}- 1 \right| \ge N^{-\alpha } \right) =O(\exp ({-}N^{1-2\alpha })). \end{aligned}$$

For $0<c< 1$ we have

$$\begin{aligned} {\mathbb {P}} \left( \sum _{i=1}^{N} Y_i < cN \right) = O( \exp (- N I(c))), \end{aligned}$$

where *I*(*y*) is the rate function of *Y*. Condition (8) ensures that $I(c)>0$ for ${\mathbb {E}} \left[ Y_1 \right] =1 \ne c$, see Dembo and Zeitouni (1994) Theorem 2.2.3.

For any $a,a' \ge 1$ one has $\left| \frac{1}{a}-\frac{1}{a'} \right| \le |a-a'|$. This yields

$$\begin{aligned} {\mathbb {P}} \left( \left| \frac{N }{\sum _{j=1}^{N} Y_j}- 1\right| \ge N^{-\alpha } \right)&= {\mathbb {P}} \left( \frac{1}{c} \left| \frac{N c }{\sum _{j=1}^{N} Y_j}- c \right| \ge N^{-\alpha } \right) \\&\le {\mathbb {P}} \left( \frac{1}{c^2} \left| \frac{\sum _{j=1}^{N} Y_j}{N}- 1\right| \ge N^{-\alpha } \right) + O( e^{ - N I(c)} )\\&= {\mathbb {P}} \left( \frac{1}{\sqrt{N}} \left| \sum _{i=1}^N (Y_i -1 ) \right| \ge c ^2 N^{\frac{1}{2}-\alpha } \right) +O( e^{ - N I(c)} ). \end{aligned}$$

Using Cramér (1938) Theorem 1, the probability on the r.h.s. can, with a suitable ${\widetilde{c}} >0$, be estimated from above by

$$\begin{aligned}&\exp \left( {\widetilde{c}} N ^{1-3\alpha } \right) \exp \left( - \frac{c^4}{2} N^{1-2\alpha }\right) (1+O(N^{-\alpha } \log N))\\&\quad = \exp \left( -\frac{c^4}{2} N^{1 - 2 \alpha }\right) (1+o(1)), \end{aligned}$$

which gives the desired result. $\square$

## Proof of the main result

Recall from (13) that we denote the order of the selection strength by $b_N= {-} \frac{\log s_N}{\log N}$. To simplify notation we will drop the subscript and simply write $b:=b_N$. As mentioned already in the sketch of the proof of Theorem 1 we assume without loss of generality that $\mathbbm {E}[Y]=1.$

The proof of the Theorem is divided into three parts, which correspond to three phases of growth for the Cannings frequency process ${\mathcal {X}}$. The initial phase is decisive: due to Proposition 1, the probability that ${\mathcal {X}}$ reaches the level $N^{b+\delta }$ for some sufficiently small $\delta$ is given by the r.h.s. of (14). Lemmas 6 and 7 then guarantee that, once having reached the level $N^{b+\delta }$, the process ${\mathcal {X}}$ reaches *N* with high probability. The proof of the Theorem is then a simple combination of these three results and the strong Markov property. Indeed, with $\tau _1,\tau _2,\tau _3$ as in Proposition 1, Lemmas 6 and 7, and with $\delta , \delta ',\varepsilon$ fulfilling the requirements specified there, the fixation probability in the l.h.s. of (14) can be rewritten as

$$\begin{aligned} {\mathbb {P}}(X_{\tau }=N)&= {\mathbb {P}}(X_{\tau _3}=N| X_{\tau _2} \ge \varepsilon N ) {\mathbb {P}}(X_{\tau _2} \ge \varepsilon N| X_{\tau _1} \ge N^{b+\delta } ) {\mathbb {P}}_1(X_{\tau _1}\ge N^{b+\delta }) \\&= (1-o(1)) (1-O(N^{-\delta '})) \frac{2 s_N}{\rho ^2} (1+o(1))\\&\quad {\sim }\frac{2 s_N}{\rho ^2}. \end{aligned}$$

### First phase: from 1 to $N^{b+\delta }$

In this section we show that as long as $X_g \le N^{b+\delta }$ the process ${\mathcal {X}}$ can be upper and lower bounded (with sufficiently high probability) by two slightly supercritical branching processes $\underline{ {\mathcal {Z}} } = ({\underline{Z}}_g)_{g\ge 0}$ and $\overline{ {\mathcal {Z}} }=({\overline{Z}}_g)_{g\ge 0}$. To construct the upper bound $\overline{ {\mathcal {Z}}}$ we take the highest per capita selective advantage, which occurs when only a single individual is beneficial. Using Lemmas 3 and 4, we will approximate the thus arising mixed binomial distribution by a mixed Poisson distribution, which leads for $\overline{ {\mathcal {Z}}}$ to the offspring distribution

29 $$\begin{aligned} \text {Pois}\left( Y_1 (1+ s_N + o(s_N))\right) , \end{aligned}$$

where $Y_1$ is the random variable figuring in (7). To arrive at the lower bounding Galton–Watson process $\underline{ {\mathcal {Z}} }$ we note that the per capita selective advantage is bounded from below by the one when $\lceil N^{b+\delta } \rceil$ beneficial individuals are present in the parent generation, as long as the process ${\mathcal {X}}$ has not reached the level $N^{b+\delta }$. Again using Lemmas 3 and 4 we will show that the offspring distribution of $\underline{\mathcal Z}$ can be chosen as the mixed binomial distribution

30 $$\begin{aligned} \text { Bin} \left( N - \lceil N^{b+\delta } \rceil , \frac{Y_1}{N } (1+ s_N + o(s_N)) \right) . \end{aligned}$$

#### Lemma 5

(Coupling with Galton–Watson processes) Let $\delta$ and $\alpha$ be such that $0<\delta < \eta$ and $\frac{1}{2}-\eta< \alpha < \frac{1}{2}$ , and put

$$\begin{aligned} \tau _1=\inf \{ g\ge 0 : X_g =0 \text { or } X_g \ge N^{b+\delta }\}. \end{aligned}$$

Then ${\mathcal {X}}$ can be defined on one and the same probability space together with two branching process $\underline{ {\mathcal {Z}}}$ and $\overline{{\mathcal {Z}}}$ with offspring distributions (30) and (29), respectively, such that for $j=1,2,\ldots$

31 $$\begin{aligned} {\mathbb {P}}( {Z}_{j \wedge \tau _1} { \wedge \lceil N^{b+\delta } } \rceil \le X_{j\wedge \tau _1}&{ \wedge \lceil N^{b+\delta } \rceil }\le {\overline{Z}}_{j\wedge \tau _1}\, \big |\, {Z}_{j-1\wedge \tau _1} \le X_{j-1\wedge \tau _1} \le {\overline{Z}}_{j-1\wedge \tau _1})\nonumber \\&\ge 1- e^{ -c' N^{1-2\alpha }} (1+o(1)), \end{aligned}$$

with $c'$ as in Lemma 4.

Applying the latter estimate *g* times consecutively yields immediately the following corollary:

#### Corollary 1

Let $\delta , \alpha , \tau _1,\underline{{\mathcal {Z}}}$ and $\overline{{\mathcal {Z}}}$ be as in Lemma 5. If $X_0 \le N^{b+\delta }$, then for all $g \in {\mathbb {N}}_0$

32 $$\begin{aligned} {\mathbb {P}}( {Z}_{g \wedge \tau _1}{ \wedge \lceil N^{b+\delta } \rceil }&\le X_{g\wedge \tau _1} { \wedge \lceil N^{b+\delta } \rceil } \le {\overline{Z}}_{g\wedge \tau _1}| {Z}_{0} \le X_{0}\le {\overline{Z}}_{0})\nonumber \\&\ge \left( 1- O(\exp (-c'N^{1-2\alpha } )) \right) ^g. \end{aligned}$$

#### Proof

*of Lemma* 5. We proceed inductively, assuming that for $g=1,2,\ldots$ we have constructed ${\mathcal {X}}$, $\overline{{\mathcal {Z}}}$ and $\underline{{\mathcal {Z}}}$ up to generation $g-1$ such that (31) holds for $j=1, \ldots , g-1$. Together with $X_g$ we will construct ${{\overline{Z}}}_g$ and ${\underline{Z}}_g$, and check the asserted probability bound for the coupling.

Given $\{X_{g-1}=k\}$ and the weights $(W_i)$ in generation $g-1$, the number of beneficial individuals $X_g$ in generation *g* has the binomial distribution (6). Aiming first at the construction of the upper bound $\overline{{\mathcal {Z}}}$, we relate (6) to (29) in terms of stochastic order. For $p, p'\ge 0$, a Bin(*N*, *p*)-distributed random variable *B* is stochastically dominated by a Pois$(Np')$-distributed random variable *P* if

33 $$\begin{aligned} e^{- p'} \le (1-p), \end{aligned}$$

see (1.21) in Klenke and Mattner (2010). Indeed, in this case the probability of the outcome zero is not larger for a Pois($p'$)-distributed random variable $P_1$ than for a Bernoulli(*p*)-distributed random variable $B_1$, which yields $B_1 \preceq P_1$, where $\preceq$ denotes the usual stochastic ordering of the random variables. Consequently

$$\begin{aligned} B{\mathop {=}\limits ^{d}} \sum _{i=1}^{N} B_i \preceq \sum _{i=1}^N P_i {\mathop {=}\limits ^{d}} P. \end{aligned}$$

with $B_i$ and $P_i$ being independent copies of $B_1$ and $P_1$, respectively. In particular, for $p\ge 0$ and $p'= p (1+N^{b +2 \delta -1})$ we have

$$\begin{aligned} e^{-p'} \le 1-p'+ (p')^2 = 1-p (1+N^{b +2 \delta -1}) + p^2 (1+N^{b +2 \delta -1})^2. \end{aligned}$$

Hence Condition (33) holds if

34 $$\begin{aligned} p (1+N^{b +2 \delta -1})^2&< N^{b +2 \delta -1}. \end{aligned}$$

Given $(W_i)$, the success probability of the binomial distribution (6) is bounded from above via

$$\begin{aligned} p:=\left( \sum _{i=1} ^{k} W_i\right) /(1-s_N). \end{aligned}$$

Thus by Lemma 3, (34) is fulfilled with probability $1- O(\exp (-c_\varepsilon N^{b+\delta }))$ with $c_\varepsilon$ as in Lemma 3. In this sense the number of beneficial offspring is dominated by a Pois$\left( N \frac{\sum _{i=1} ^{k} W_i}{(1-s_N)} (1+N^{b+2\delta -1}) \right)$-distributed random variable with high probability. Applying Lemma 4 yields that with probability $1-\exp (-c' N^{1-2\alpha })(1+o(1))$ the following chain of inequalities is valid:

$$\begin{aligned} N \frac{\sum _{i=1} ^{X_{g-1}} W_i}{(1-s_N)}(1+N^{b+2\delta -1})&\le \frac{\sum _{i=1} ^{X_{g-1}} Y_i}{(1-s_N)} (1+N^{b+2\delta -1}) (1+N^{-\alpha }) \\&=\!\sum _{i=1}^{X_{g-1}} Y_i (1+s_N+o(s_N)) \le \! \sum _{i=1}^{{\overline{Z}}_{g-1}} Y_i (1+s_N+o(s_N)). \end{aligned}$$

In this way ${\mathcal {X}}$ can be coupled with a branching process $\overline{ {\mathcal {Z}} }$ with a mixed Poisson offspring distribution of the form (29).

The lower bound also uses a comparison with a Galton–Watson process, now with a mixed binomially distributed offspring distribution:

Number the individuals in generation $g-1$ by $(i,g-1)$, with $(1,g-1), \ldots , (X_{g-1},g-1)$, being the beneficial individuals. Given ${\mathscr {W}}$, we use a sequence of coin tossings to determine which of the individuals from generation *g* are the children of $(i,g-1)$. The first *N* tosses determine which individuals are the children of $(1,g-1)$. Denoting the number of these children by $\omega _1^{g-1}$, the next $N-\omega _1^{g-1}$ tosses (with an updated success probability) determine which individuals are the children of $(2,g-1)$, etc. Observe that as long as $X_{g-1} \le N^{b+\delta }$, and given ${\mathscr {W}}$ and $\sum _{\ell =1}^{i-1}\omega _\ell ^{(g-1)}=:h$, then $\omega _i^{(g-1)}$ for $i \le X_{g-1}$ has distribution

35 $$\begin{aligned} \text { Bin} \left( N-h , \frac{W_i}{\sum _{\ell = i}^{X_{g-1}}W_\ell + (1-s_N) \sum _{\ell = X_{g-1} +1}^{N} W_\ell } \right) . \end{aligned}$$

Note that the success probability in (35) can be estimated from below by

$$\begin{aligned} \frac{W_i}{\sum _{\ell =1}^{X_{g-1} } W_\ell + (1-s_N) \sum _{\ell = X_{g-1} +1 }^N W_\ell } = \frac{W_i}{1-s_N +s_N \sum _{\ell =1}^{X_{g-1}} W_\ell }. \end{aligned}$$

As long as $X_{g-1} \le \lceil N^{b+\delta } \rceil$, Lemma 3 ensures that for $\varepsilon > 0$

36 $$\begin{aligned} {\frac{W_i}{1-s_N +s_N \sum _{j=1}^{X_{g-1}} W_j}} \ge \frac{W_i}{1-s_N + (1+\varepsilon )N^{\delta -1}} \end{aligned}$$

with probability $1-O(\exp (-c_\varepsilon N^{b+\delta }))$. Lemma 4, in turn, yields that the r.h.s. of (36) is bounded from below by

$$\begin{aligned} \frac{Y_i}{N (1-s_N + (1+\varepsilon )N^{\delta -1})} (1+ N^{-\alpha }) = \frac{Y_i}{N } (1+ s_N +o(s_N)) \end{aligned}$$

with probability at least $1-\exp (-c' N^{1-2\alpha })(1+o(1))$.

Thus, if $\omega _1^{(g-1)}+\dots +\omega _{i-1}^{(g-1)}=h \le { \lceil N^{b+\delta } \rceil }$, then the distribution of $\omega _i^{(g-1)}$ specified in (35) is bounded from below by

$$\begin{aligned} \text {Bin}\left( N-\lceil N^{b+\delta } \rceil , \frac{W_i}{1-s_N + (1+\varepsilon )N^{\delta -1}} \right) \end{aligned}$$

with probability $$1-O(\exp (-c_\varepsilon N^{b+\delta }))$$.

If $\omega _1^{(g-1)}+\dots +\omega _{i-1}^{(g-1)}=h > \lceil N^{b+\delta } \rceil$, then we have ${Z}_{g \wedge \tau _1} \wedge \lceil N^{b+\delta } \rceil \le X_{g\wedge \tau _1} \wedge \lceil N^{b+\delta } \rceil$. Consequently ${\mathcal {X}}$ can be coupled with a Galton–Watson process $\underline{{\mathcal {Z}}}$ with offspring distribution of the form (30) such that also the lower estimate in (32) is fulfilled. This completes the proof of Lemma 5. $\square$

We are now ready to prove that ${\mathcal {X}}$ reaches the level $N^{b+\delta }$ with probability $\frac{2s_N}{\rho ^2} (1+o(1))$.

#### Proposition 1

(Probability to reach the critical level) Assume Conditions (7), (8) and (12) are fulfilled and define $\tau _1 = \inf \{ g \ge 0 : X_g \ge N^{b+\delta } \text { or } X_g=0 \}$ with $0<\delta < \eta$, then

37 $$\begin{aligned} {\mathbb {P}}( X_{\tau _1} \ge N^{b+\delta } ) = \frac{2 s_N}{\rho ^2 }(1+o(1)). \end{aligned}$$

#### Proof

We use the couplings of ${\mathcal {X}}$ with the slightly supercritical branching processes $\underline{ {\mathcal {Z}} }$ and $\overline{ {\mathcal {Z}} }$ from Corollary 1 and show that both processes reach the level $N^{b+\delta }$ with probability $\frac{2s_N}{\rho ^2}(1 + o(1))$. Let $\delta '>0$ and *E* be the event that the stochastic ordering between $\underline{{\mathcal {Z}}},{\mathcal {X}}$ and $\overline{ {\mathcal {Z}}}$ holds until generation $n_0=\lceil N^{b+\delta '} \rceil$, that is

$$\begin{aligned} E= \{{\underline{Z}}_0 { \wedge \lceil N^{b+\delta } \rceil } \le X_0 { \wedge \lceil N^{b+\delta } \rceil } \le \overline{ Z}_0,\ldots ,{\underline{Z}}_{n_0} { \wedge \lceil N^{b+\delta } \rceil } \le X_{n_0} { \wedge \lceil N^{b+\delta } \rceil } \le \overline{ Z}_{n_0} \}. \end{aligned}$$

We show below that the stopping time $\tau _1$ fulfils

38 $$\begin{aligned} {\mathbb {P}}(\tau _1 \ge \lceil N^{b+\delta '} \rceil ) = o(s_N). \end{aligned}$$

For some *g* that is polynomially bounded in *N*, the r.h.s. of (32) is bounded from above by $1-o(s_N)$. Thus, combining Corollary 1 and (38) we deduce

$$\begin{aligned} {\mathbb {P}}(E, \tau _1 \le \lceil N^{b+\delta '} \rceil ) =1-o(s_N). \end{aligned}$$

We are now going to bound (37) from above by estimating the corresponding probability for $\overline{\mathcal Z}$ and the stopping time ${\overline{\tau }}_1 = \inf \{ g \ge 0 : {\overline{Z}}_g \ge N^{b+\delta } \text { or } {\overline{Z}}_g=0 \}$. More precisely,

$$\begin{aligned} {\mathbb {P}}( X_{\tau _1} \ge N^{b+\delta } )&= {\mathbb {P}}( X_{\tau _1} \ge N^{b+\delta }, \tau _1 \le \lceil N^{b+\delta '} \rceil ,E )+ o(s_N) \\&\le {\mathbb {P}}( {\overline{Z}}_{{\overline{\tau }}_1} \ge N^{b+\delta }, \tau _1 \le \lceil N^{b+\delta '} \rceil ,E )+ o(s_N)\\&\le {\mathbb {P}}( {\overline{Z}}_{{\overline{\tau }}_1} \ge N^{b+\delta })+ o(s_N). \end{aligned}$$

To obtain an upper bound for the probability of $\overline{{\mathcal {Z}} }$ to reach the level $N^{b+\delta }$ it suffices to estimate the survival probability of $\overline{{\mathcal {Z}} }$. For notational simplicity let us write $\{ \overline{ {\mathcal {Z}}} \text { survives} \}$ for the event $\{ \forall g\ge 0: {\overline{Z}}_g >0 \}$ and similarly $\{ \overline{ {\mathcal {Z}}} \text { dies out} \}$ for the event $\{ \exists g \ge 0 : {\overline{Z}}_g =0 \}$. We have

$$\begin{aligned}&{\mathbb {P}}_1 \left( {\overline{Z}}_{{\overline{\tau }}_1} \ge N^{b+\delta } \right) \\&\quad \le {\mathbb {P}}_1 \left( {\overline{Z}}_{{\overline{\tau }}_1} \ge N^{b+ \delta }|\overline{{\mathcal {Z}}} \text { survives }\right) {\mathbb {P}}_1( \overline{{\mathcal {Z}}} \text { survives }) +{\mathbb {P}}_1 \left( {\overline{Z}}_{{\overline{\tau }}_1} \ge N^{b+\delta }|\overline{{\mathcal {Z}}} \text { dies out }\right) \\&\quad \le {\mathbb {P}}_1( \overline{{\mathcal {Z}}} \text { survives }) + {\mathbb {P}} \left( \text {all } \lceil N^{b + \delta } \rceil \text { individuals die out} \right) \\&\quad ={\mathbb {P}}_1( \overline{{\mathcal {Z}}} \text { survives })+ (1-{{\mathbb {P}}_1( \overline{{\mathcal {Z}}} \text { survives })}) ^{ \lceil N^{b+ \delta } \rceil }. \end{aligned}$$

The survival probability of $\overline{{\mathcal {Z}}}$ will now be estimated by means of (18). To this purpose we calculate the expectation and variance of the offspring distribution (29).

The expectation is $1+s_N +o(s_N)$ and the variance is given by

$$\begin{aligned}&\text {Var}\left( \text {Pois}\left( Y_1 (1+s_N+o(s_N) ) \right) \right) \\&\quad =\text {Var}\left( {\mathbb {E}} \left[ \text {Pois}\left( Y_1(1+s_N+o(s_N) ) \right) \Big | Y_1 \right] \right) \\&\qquad +{\mathbb {E}} \left[ \text {Var}\left( \text {Pois} \left( Y_1(1+s_N+o(s_N) ) \right) \Big | Y_1 \right) \right] \\&\quad =\text {Var}\left( Y_1 (1+s_N+o(s_N) ) \right) +{\mathbb {E}} \left[ Y_1(1+s_N+o(s_N) ) \right] \\&\quad = (1+s_N+o(s_N))^2 \text {Var}\left( Y_1 \right) +1+s_N+o(s_N)= \rho ^2 (1+o(1)). \end{aligned}$$

Equation (18) yields that the survival probability of the process $\overline{{\mathcal {Z}}}$ is given by $\frac{2 s_N}{\rho ^2}(1+o(1))$. The lower bound in (37) follows by similar arguments by considering the process $\underline{{\mathcal {Z}}}$ instead.

It remains to show (38). Define ${\overline{\tau }}^{(0)}$ and ${\underline{\tau }}^{(\text {u})}$ as the stopping times that the process $\overline{{\mathcal {Z}}}$ reaches 0 and the process $\underline{{\mathcal {Z}}}$ reaches the upper bound $N^{b+\delta }$, respectively, i.e.

$$\begin{aligned} {\overline{\tau }}^{(0)} = \inf \{ g \ge 0 : {\overline{Z}}_g =0 \}, \qquad {\underline{\tau }}^{(\text {u})}=\inf \{ g \ge 0 : {\underline{Z}}_g \ge N^{b+\delta } \}, \end{aligned}$$

with the convention that the infimum over an empty set is infinity. Then

39 $$\begin{aligned} {\mathbb {P}}(\tau _1 \ge \lceil N^{b+\delta '} \rceil )&\le {\mathbb {P}}(\tau _1 \ge \lceil N^{b+\delta '} \rceil , E )+ {\mathbb {P}}(E^c ) \nonumber \\&\le {\mathbb {P}}({\underline{\tau }}^{(\text {u})} \ge \lceil N^{b+\delta '} \rceil , {\overline{\tau }}^{(0)} \ge \lceil N^{b+\delta '} \rceil ,E)+ {\mathbb {P}}(E^c ) \nonumber \\&= {\mathbb {P}}({\underline{\tau }}^{(\text {u})} \ge \lceil N^{b+\delta '} \rceil , {\overline{\tau }}^{(0)} \ge \lceil N^{b+\delta '} \rceil ,E,\underline{ {\mathcal {Z}} } \text { dies out } ) \nonumber \\&\quad \, +{\mathbb {P}}({\underline{\tau }}^{(\text {u})} \ge \lceil N^{b+\delta '} \rceil , {\overline{\tau }}^{(0)} \ge \lceil N^{b+\delta '} \rceil ,E,\underline{ {\mathcal {Z}} } \text { survives } ) + {\mathbb {P}}(E^c ) \nonumber \\&={\mathbb {P}}({\underline{\tau }}^{(\text {u})} \ge \lceil N^{b+\delta '} \rceil , {\overline{\tau }}^{(0)} \ge \lceil N^{b+\delta '} \rceil ,E,\underline{ {\mathcal {Z}} } \text { dies out } )\nonumber \\&\quad \, +O(e^{-c' N^{\frac{\delta '}{2}} }) + O(N^{b+\delta '} e^{- \frac{1}{2}N^{1-2\alpha })}, \end{aligned}$$

by an application of Lemma 1 and Corollary 1 and $\alpha < \frac{1}{2}$ as defined there. To keep the notation simple we denote by $e_N$ terms of the order $\exp ({-N^{c}})$ for some $c>0.$ Proceeding with (39) we obtain40again by an application of Lemma 1. Note that

$$\begin{aligned}&{\mathbb {P}}( \underline{ {\mathcal {Z}} } \text { dies out }, \overline{ {\mathcal {Z}} } \text { survives}, E ) + {\mathbb {P}}( \underline{ {\mathcal {Z}} } \text { survives }, \overline{ {\mathcal {Z}} } \text { survives}, E )\\&\quad = {\mathbb {P}}( \overline{ {\mathcal {Z}} } \text { survives}, E) = \frac{2s_N}{\rho ^2}(1+o(1)). \end{aligned}$$

In order to show that (40) is $o(s_N)$ it suffices to prove that

$$\begin{aligned} {\mathbb {P}}( \underline{ {\mathcal {Z}} } \text { survives }, \overline{ {\mathcal {Z}} } \text { survives}, E )= \frac{2s_N}{\rho ^2} (1+o(1)). \end{aligned}$$

Considering again the event $\{ {\underline{\tau }}^{(u)} \le \lceil N^{b+\delta '} \rceil \}$ and applying (18) one obtains

$$\begin{aligned} {\mathbb {P}}( \underline{ {\mathcal {Z}} } \text { survives }, \overline{ {\mathcal {Z}} } \text { survives}, E )&= {\mathbb {P}}( \underline{ {\mathcal {Z}} } \text { survives }, \overline{ {\mathcal {Z}} } \text { survives}, E , {\underline{\tau }}^{(u)} \le \lceil N^{b+\delta '} \rceil ) + e_N \\&={\mathbb {P}}( \underline{ {\mathcal {Z}} } \text { survives },E , {\underline{\tau }}^{(u)} \le \lceil N^{b+\delta '} \rceil ) + e_N, \end{aligned}$$

since the events *E* and $\{ {\underline{\tau }}^{(u)} \le \lceil N^{b+\delta '} \rceil )\}$ imply that ${\overline{Z}}_g \ge N^{b+\delta }$ for some $g \le N^{b+\delta '}$ and the probability for $\overline{ {\mathcal {Z}} }$ to die out after reaching $N^{b+\delta }$ is $(1-\frac{2s_N}{\rho ^2}(1+o(1)))^{N^{b+\delta }}=e_N$. One more application of (18) yields

$$\begin{aligned} {\mathbb {P}}( \underline{ {\mathcal {Z}} } \text { survives },E , {\underline{\tau }}^{(u)} \le \lceil N^{b+\delta '} \rceil )= \frac{2s_N}{\rho ^2}(1+o(1)), \end{aligned}$$

which finishes the proof. $\square$

### Second phase: from $N^{b+\delta }$ to $\varepsilon N$

In this section we show that ${\mathcal {X}}$, once having reached the level $N^{b+\delta }$, will reach the level $\varepsilon N$ with probability tending to 1 as $N\rightarrow \infty$.

#### Lemma 6

(From $N^{b+\delta }$ to $\varepsilon N$ with high probability) Assume $X_0 \ge N^{b+\delta }$ with $0<\delta <\eta$, let $0<\varepsilon <\frac{\delta }{2-2\eta -\delta }$ and define the stopping time

$$\begin{aligned}\tau _2 =\inf \{g \ge 0 : X_g \notin \{1,2,\ldots , \lfloor \varepsilon N \rfloor \}\}. \end{aligned}$$

Then there exists some $\delta '>0$ such that

$$\begin{aligned} {\mathbb {P}} \left( X_{\tau _2} \ge \varepsilon N \right) = 1-O(N^{-\delta '}). \end{aligned}$$

#### Proof

By monotonicity it is enough to prove the claim for $X_0 = \lceil N^{b+\delta } \rceil$. By definition we have

41 $$\begin{aligned} {\mathscr {L}} (X_{g+1}|X_g)=\text {Bin}\left( N,\frac{\sum _{i=1}^{X_g} W_i }{\sum _{i=1}^{X_g} W_i+(1-s_N) \sum _{i=X_g +1}^{N} W_i} \right) . \end{aligned}$$

Next we lower-bound ${\mathcal {X}}$ by the process $\widetilde{{\mathcal {X}}} = ({\widetilde{X}}_g)_{g\ge 0}$, ${\widetilde{X}}_0 = X_0$, with conditional distribution

42 $$\begin{aligned} {\mathscr {L}} ({\widetilde{X}}_{g+1}|{\widetilde{X}}_g)=\text {Bin}\left( N,\frac{\sum _{i=1}^{{\widetilde{X}}_g} W_i}{ 1-s_N \sum _{i=\varepsilon N +1}^{N} W_i} \right) \end{aligned}$$

as long as ${\widetilde{X}}_g \le \varepsilon N$. If ${\widetilde{X}}_g > \varepsilon N$ we assume that ${\widetilde{X}}_{g+1}$ is distributed as a slightly supercritical branching process with Pois$(Y_1 q_N)$ distributed offspring, where

43 $$\begin{aligned} q_N = N {\mathbb {E}}\left[ \frac{W_1}{1-s_N\sum _{i=\varepsilon N+1}^N W_i}\right] . \end{aligned}$$

We will see that by this definition in each generation the expectation of $\widetilde{{\mathcal {X}}}$ increases by the factor $q_N$, see (45) and (46). The generation-wise increase of the variance conditioned on the current state can be estimated from above by a factor $\rho ^2(1+ o(1))$, see (45) and (47), leading to an iterative estimate on the variance of the form (48). As long as $X_g \ge {\widetilde{X}}_g$, the success probability in the mixed Binomial distribution on the r.h.s. of (41) dominates the corresponding one on the r.h.s. of (42). Consequently, starting ${\widetilde{X}}$ and *X* both in $\lfloor N^{b+\delta } \rfloor$ we can couple them, such that ${\widetilde{X}}_g \le X_g$ as long as ${\widetilde{X}}$ did not cross the level $\varepsilon N$. In particular, we have for the stopping time $\widetilde{\tau } = \inf \{ g \ge 0 : \widetilde{X}_g \notin \{1,2, \dots , \lfloor \varepsilon N \rfloor \} \}$

44 $$\begin{aligned} {\mathbb {P}}\big (X_{\tau } \ge \varepsilon N\big ) \ge \mathbb P\big ({\widetilde{X}}_{{\widetilde{\tau }}} \ge \varepsilon N\big ). \end{aligned}$$

To show ${\mathbb {P}} \left( {\widetilde{X}}_{{\widetilde{\tau }}} \ge \varepsilon N \right) = 1-O(N^{-\delta '})$ we will estimate the first and second moment of ${\widetilde{X}}_{g_0}$ for a suitably chosen $g_0 \in {\mathbb {N}}$ and then use Chebyshev’s inequality to show that ${\widetilde{X}}_{g_0}$ is above $\varepsilon N$ with sufficiently high probability. For this purpose we consider *m*(*x*) and *v*(*x*), the one-step conditional expectation and variance of ${\widetilde{X}}$ at $x \in {\mathbb {N}}$, that is

$$\begin{aligned} m(x)={\mathbb {E}} \left[ {\widetilde{X}}_1|{{\widetilde{X}}}_0=x \right] , \qquad v(x)=\text {Var}\left( {\widetilde{X}}_1|{{\widetilde{X}}}_0=x \right) . \end{aligned}$$

From the definition of $\widetilde{{\mathcal {X}}}$ as a branching process above $\varepsilon N$ we have for $x >\varepsilon N$

45 $$\begin{aligned} m(x)=q_N x, \qquad v(x)= \rho ^2 x (1+o(1)). \end{aligned}$$

Next we show that *m*(*x*) and *v*(*x*) fulfil relations similar to (45) also for $x \le \varepsilon N$, which will allow to estimate the expectation and the variance of ${{\widetilde{X}}}_{g_0}$.

For $x \le \varepsilon N$ we have due to (42)

$$\begin{aligned} m(x)&= N {\mathbb {E}} \left[ \frac{\sum _{i=1}^{x} W_i}{ 1-s_N \sum _{i=\varepsilon N +1}^{N} W_i} \right] \\&= N {\mathbb {E}} \left[ \sum _{i=1}^{x} W_i \left( 1+s_N \sum _{i=\varepsilon N +1}^{N} W_i+ O(s_N^2) \right) \right] \\&=x( 1+s_N (1-\varepsilon ) N^2 {\mathbb {E}} \left[ W_1 W_{\varepsilon N +1} \right] + O(s_N^2))\\&={x\left( 1+s_N (1-\varepsilon )(1+O(N^{-1})) + O(s_N^2)\right) }\\&= x \left( 1+(1-\varepsilon ) s_N + O(s_N^2)\right) . \end{aligned}$$

In the penultimate equality we used ${\mathbb {E}} \left[ W_1W_2 \right] = \frac{1}{N^2} + O(N^{-3})$ which results from the fact

$$\begin{aligned} 1={\mathbb {E}} \left[ \left( \sum _{i=1}^{N}W_i\right) ^2 \right] =N {\mathbb {E}} \left[ W_1^2 \right] + N(N-1){\mathbb {E}} \left[ W_1W_2 \right] \end{aligned}$$

and (11). Consequently, we have for all $x\in {\mathbb {N}}$, recalling (43),

46 $$\begin{aligned} m(x)= x q_N = x \left( 1+(1-\varepsilon ) s_N + O(s_N^2)\right) . \end{aligned}$$

Next we analyse *v*(*x*), again for $x \le \varepsilon N$. In view of (42), a decomposition of the variance gives

$$\begin{aligned} v(x)&=\text {Var}\left( N \frac{\sum _{i=1}^{x} W_i}{1-s_N \sum _{i=\varepsilon N +1}^N W_i} \right) \\&\quad + {\mathbb {E}} \left[ N \frac{\sum _{i=1}^{x} W_i}{1-s_N \sum _{i=\varepsilon N +1}^N W_i} \left( 1- \frac{\sum _{i=1}^{x} W_i}{1-s_N \sum _{i=\varepsilon N +1}^N W_i}\right) \right] \\&\le {\mathbb {E}} \left[ \left( N \frac{\sum _{i=1}^{x} W_i}{1-s_N \sum _{i=\varepsilon N +1}^N W_i}\right) ^2 \right] - {\mathbb {E}} \left[ N \frac{\sum _{i=1}^{x} W_i}{1-s_N \sum _{i=\varepsilon N +1}^N W_i} \right] ^2\\&\quad + {\mathbb {E}} \left[ N \frac{\sum _{i=1}^{x} W_i}{1-s_N} \right] \\&{ \le {\mathbb {E}} \left[ \left( N \frac{\sum _{i=1}^{x} W_i}{1-s_N}\right) ^2 \right] - {\mathbb {E}} \left[ N\sum _{i=1}^{x} W_i \right] ^2 + {\mathbb {E}} \left[ N \frac{\sum _{i=1}^{x} W_i}{1-s_N} \right] . } \end{aligned}$$

Because of the negative correlation of the $W_i$, the sum of the first and the second term is not larger than $x N^2 \text {Var}\left( W_1 \right)$, which because of (11) is $\le x (\rho ^2 -1) + O(N^{-1})$. Since the third term is $x(1 + O(s_N))$, we have for all $x \le \varepsilon N$

47 $$\begin{aligned} v(x) \le \rho ^2 x (1+o(1)). \end{aligned}$$

Combining (46) and (47) allows us to estimate the variance $\text {Var}\left( {\widetilde{X}}_g \right)$ for $g\in {\mathbb {N}}$, again by decomposing the variance:

48 $$\begin{aligned} \text {Var}\left( {\widetilde{X}}_g \right)&= \text {Var}\left( {\mathbb {E}} \left[ {\widetilde{X}}_g|{\widetilde{X}}_{g-1} \right] \right) +{\mathbb {E}} \left[ \text {Var}\left( {\widetilde{X}}_g|{\widetilde{X}}_{g-1} \right) \right] \nonumber \\&= \text {Var}\left( m({\widetilde{X}}_{g-1}) \right) + {\mathbb {E}} \left[ v({\widetilde{X}}_{g-1}) \right] \nonumber \\&\le q_N^2 \text {Var}\left( {\widetilde{X}}_{g-1} \right) + \rho ^2 {\mathbb {E}} \left[ {\widetilde{X}}_{g-1} \right] (1+o(1)) \nonumber \\&=q_N^2 \text {Var}\left( {\widetilde{X}}_{g-1} \right) + \rho ^2 q_N^{g-1} {\widetilde{X}}_0 (1+o(1)). \end{aligned}$$

Iterating this argument yields

$$\begin{aligned} \text {Var}\left( {\widetilde{X}}_{g} \right)&=\rho ^2 {\widetilde{X}}_0 q_N^{g-1} \sum _{j=0}^{g-1} q_N^j (1+o(1)) \\&= \rho ^2 {\widetilde{X}}_0 q_N^{g-1} \frac{q_N^g-1}{q_N-1} (1+o(1)). \end{aligned}$$

Choose the minimal $g_0 \in {\mathbb {N}}$ such that $2 \varepsilon N \le {\mathbb {E}} \left[ {\widetilde{X}}_{g_0} \right] = q_N^{g_0} X_0$, which yields recalling the initial condition $X_0 = \lceil N^{b+\delta }\rceil$

$$\begin{aligned} g_0= \left\lceil \frac{\log (2 \varepsilon N X_0^{-1})}{\log q_N} \right\rceil = \left\lceil \frac{\log (2 \varepsilon N^{1-b-\delta })}{(1-\varepsilon ) s_N + O(s_N^2)} \right\rceil . \end{aligned}$$

Applying Chebyshev’s inequality with ${\widetilde{X}}_0 = X_0$, we obtain

$$\begin{aligned} {\mathbb {P}}\left( |{\widetilde{X}}_{g_0} - {\mathbb {E}} \left[ {\widetilde{X}}_{g_0} \right] | \ge \varepsilon N \right)&\le \frac{\rho ^2 {\widetilde{X}}_0 q_N^{g_0-1} \frac{q_N^{g_0}-1}{q_N-1} (1+o(1)) }{\varepsilon ^2 N^2}\\ \\&\le \frac{\rho ^2 N^{b+\delta } q_N^{2g_0} \frac{N^b}{(1-\varepsilon )} (1+o(1))}{\varepsilon ^2 N^2}\\&= \frac{\rho ^2}{\varepsilon ^2 (1-\varepsilon )} N^{2b+\delta -2} (1+(1-\varepsilon ) s_N + O(s_N^2))^{2g_0} (1+o(1)) \\&\le c_{\rho ,\varepsilon } N^{2b+\delta -2} \exp ( 2g_0 s_N (1+O(s_N))) (1+o(1))\\&\le c_{\rho ,\varepsilon } N^{2b+\delta -2} N^{\frac{2}{1-\varepsilon } (1-b-\delta )} (1+o(1)) \\&= O(N^{-\delta '}), \end{aligned}$$

for some small $\delta '>0$ due to the assumptions on $\varepsilon$.

Since ${\mathbb {E}} \left[ {\widetilde{X}}_{g_0} \right] \ge 2\varepsilon N$, this implies

$$\begin{aligned} {\mathbb {P}} \left( {\widetilde{X}}_{{\widetilde{\tau }}} \ge \varepsilon N \right) \ge {\mathbb {P}}({\widetilde{X}}_{g_0} \ge \varepsilon N ) \ge 1- O(N^{-\delta '}) \end{aligned}$$

and due to (44) this finishes the proof. $\square$

### Third phase: from $\varepsilon N$ to *N*

Lemma 7 below concerns the last step of the proof, showing that once the process ${\mathcal {X}}$ has reached the level $\lfloor {\varepsilon N} \rfloor$, it goes to fixation with high probability. Our proof relies on a representation of the fixation probability of ${\mathcal {X}}$ in terms of (a functional of) the equilibrium state $A_{\mathrm{eq}}:=A_{\mathrm{eq}}^{(N)}$ of the counting process $\mathcal A:= {\mathcal {A}}^{(N)}=(A_m)_{ m \ge 0}$ of the *potential ancestors* in the time discrete Cannings ancestral selection graph as provided by Boenkost et al. (2021). The process ${\mathcal {A}}^{(N)}$ is called *Cannings ancestral selection process (CASP)* in Boenkost et al. (2021); for fixed *N*, it is a recurrent, [*N*]-valued Markov chain whose transition probabilities are specified in Boenkost et al. (2021) Section 2.3.

Theorem 3.1 and Formula (3.2) (see also Corollary 3.3) in Boenkost et al. (2021) provide the following sampling duality representation of the fixation probability of ${\mathcal {X}}$ when started with *k* individuals:

49 $$\begin{aligned} {\mathbb {P}}_k({\mathcal {X}} \text{ eventually } \text{ hits } N) = 1-\mathbb E\left[ \frac{(N-k)(N-k-1)\cdots (N-k-A_{\mathrm{eq}}+1)}{N(N-1)\cdots (N-A_{\mathrm{eq}}+1)}\right] . \end{aligned}$$

Intuitively, this says that ${\mathcal {X}}$ goes extinct if and only if a random sample of (random) size $A_{\mathrm{eq}}$, drawn without replacement from the population of size *N*, avoids the *k* beneficial individuals.

Formula (49) implies

50 $$\begin{aligned} {\mathbb {P}}_{\lceil {\varepsilon N} \rceil }({\mathcal {X}} \text{ eventually } \text{ hits } N) \ge 1-{\mathbb {E}} \left[ (1-\varepsilon )^{A_{\mathrm{eq}}^{(N)}}\right] . \end{aligned}$$

The representation of the transition probabilities of ${\mathcal {A}}$ in Boenkost et al. (2021) Section 2.3 in terms of two half steps yields that for fixed *N* CASPs with different selection parameters can be coupled in such a way that $A_{\mathrm{eq}}^{(N)}$ is increasing in $s_N$. Take a sequence $({\tilde{s}}_N)$ satisfying ${\tilde{s}}_N \le s_N$ and Condition (1.2) in Boenkost et al. (2021), i.e.

$$\begin{aligned} N^{-1+\eta } \le {\tilde{s}}_N \le N^{-2/3+\eta }. \end{aligned}$$

Let ${\tilde{A}}_{\mathrm{eq}}^{(N)}$ be the equilibrium state belonging to ${\tilde{s}}_N$ (and to the same Dirichlet-type paintbox as that of ${\mathcal {X}}$). The central limit result Boenkost et al. (2021), Corollary 6.10, implies that ${\tilde{A}}_{\mathrm{eq}}^{(N)}\rightarrow \infty$ in probability as $N\rightarrow \infty$. Because of the just mentioned monotonicity in the selection coefficient, the same convergence holds true for the sequence $\left( A_{\mathrm{eq}}^{(N)}\right)$. The following lemma is thus immediate from (50) and dominated convergence:

#### Lemma 7

(From $\varepsilon N$ to *N* with high probability) Let ${\mathcal {X}}$ be a Cannings frequency process with $X_0= k \ge \varepsilon N$ for some $0<\varepsilon <1/2$. Assume that Conditions (7), (8) and (12) are fulfilled. Define $\tau _3: =\inf \{g \ge 0: X_g \in \{0,N \} \}$. Then

$$\begin{aligned} {\mathbb {P}}_k(X_{\tau _3} =N) = 1-o(1). \end{aligned}$$

## Discussion

The analysis of fixation probabilities of slightly beneficial mutants is at the heart of population genetics; some seminal and more modern references are given in the Introduction. Our main result concerns Haldane’s asymptotics (3) for the fixation probability in a regime of moderate selection.

Our framework is that of Cannings models with selection (as reviewed in Sect. 2), where the corresponding neutral genealogies are assumed to belong to the domain of attraction of Kingman’s coalescent. This class of models is motivated by seasonal reproduction cycles in which within each season a large number of offspring is generated but only a comparatively small number (concentrated around a carrying capacity *N*) of randomly sampled offspring survive to the next season. In this setting it is reasonable to approximate sampling without replacement by sampling with replacement. Thus, under the assumption of neutrality, the probability that the *j*-th offspring that survives till the next generation is a child of parent *i* is approximately given by the random weight

$$\begin{aligned} W_i=\frac{Y_i}{\sum _{\ell =1}^{N} Y_{\ell }}, \end{aligned}$$

where $Y_1,\dots ,Y_N$ are the sizes of (potential one-generation) offspring of parents $1,\dots ,N$. These sizes are assumed to be independent and identically distributed in the present paper, leading to the concept of weights *of Dirichlet type*. The subsequent generation then arises by a multinomial sampling with random weights, and to add selection the weights of wildtype parents are decreased by the factor $(1-s_N)$. For a closely related model with a specific distribution of $Y_i$ (and sampling without replacement) in the context of Lenski’s long-term evolution experiment see González Casanova et al. (2017) and Baake et al. (2019).

We prove Haldane’s asymptotics in the case of *moderately strong selection*, see Theorem 1, in which the selection strength $s_N$ obeys

$$\begin{aligned} N^{-\frac{1}{2}+\eta } \le s_N \le N^{-\eta } \end{aligned}$$

for some $\eta >0$ and a large population size *N*. In the companion paper Boenkost et al. (2021) the range of *moderately weak selection* was considered, i.e. in the case

$$\begin{aligned} N^{-1+\eta } \le s_N \le N^{-\frac{1}{2} -\eta } \end{aligned}$$

for some $\eta >0.$ Since $s_N \gg N^{-1}$ in the regime of moderate selection, selection acts in this case on a faster timescale than genetic drift.

In Boenkost et al. (2021) an ancestral selection graph for the just described class of Cannings models with selection was defined, and it was shown that the fixation probability $\pi _N$ is equal to the expeced value ${\mathbb {E}} \left[ \frac{A_{\mathrm{eq}}^{(N)}}{N}\right]$, where $A_{\mathrm{eq}}^{(N)}$ is the number of lines of the ancestral selection graph in its equilibrium. While we could analyse directly the asymptotics of that quantity in the regime of moderately weak selection, we were facing too large fluctuations of $A_{\mathrm{eq}}^N$ in the regime of moderately strong selection in order to be successful with this approch. Conversely, it turned out that the classical idea of branching process approximation is suitable precisely in that latter regime.

For highly skewed offspring distributions an asymptotics for the fixation probability arises which is different from (3). In cases where the neutral genealogy is attracted by a Beta$(2-\alpha ,\alpha )$-coalescent, Okada and Hallatschek (2021) argue that the fixation probability is proportionally to $s_N^{\frac{1}{\alpha -1}}$, if $1\gg s_N \gg N^{-(\alpha -1)}$. Thus the probability of fixation is substantially smaller than in Haldane’s asymptotics, which is reasonable since the offspring variance is diverging as $N\rightarrow \infty$. Notably, since the evolutionary timescale of Cannings models in the domain of attraction of Beta-coalescents is of the order $N^{\alpha -1}$, the case $1\gg s_N \gg N^{-(\alpha -1)}$ corresponds to the regime of moderate selection; note also that the case of coalescents being in the domain of attraction of a Kingman coalescent corresponds formally to $\alpha =2$.
